# New Hampshire Veterinary Medical Association

**Published:** 1896-10

**Authors:** L. E. Tuttle

**Affiliations:** Secretary pro tem.


### NEW HAMPSHIRE VETERINARY MEDICAL ASSOCIATION.

THE twelfth meeting was held at Concord, September 1, 1896, with President Dr. Lilico in the chair. Drs. Lilico, Russell, Wilkinson, Macguire, Abbott, and Tuttle responded to roll-call. Dr. Ebbitt, of Omaha, Neb., was present as guest. A letter from the Pasteur Memorial Fund was read, and it was voted to appropriate $5 from the treasury for this purpose. Dr. Lilico, of the Legislative Committee, stated that the committee had no report as yet, but would be ready at the December meeting. Dr. Lilico read a most interesting paper on "Colic," which was followed by discussion. Dr. Ebbitt, of Nebraska, kindly offered a few remarks and brought out new points which were very interesting. The meeting adjourned at 1.30 P.M., until December.

L. E. TUTTLE, M.D.V.,
Secretary *pro tem.*

